# Neutropenic Diet Cannot Reduce the Risk of Infection and Mortality in Oncology Patients With Neutropenia

**DOI:** 10.3389/fonc.2022.836371

**Published:** 2022-03-09

**Authors:** Yimei Ma, Xiaoxi Lu, Hanmin Liu

**Affiliations:** ^1^Department of Pediatrics, West China Second University Hospital, Sichuan University, Chengdu, China; ^2^Key Laboratory of Birth Defects and Related Diseases of Women and Children (Sichuan University), Ministry of Education, Chengdu, China; ^3^Department of Pediatric Pulmonology and Immunology, West China Second University Hospital, Sichuan University, Chengdu, China; ^4^Key Laboratory of Chronobiology (Sichuan University), National Health Commission of China, Chengdu, China; ^5^The Joint Laboratory for Lung Development and Related Diseases of West China Second University Hospital, Sichuan University and School of Life Sciences of Fudan University, West China Institute of Women and Children’s Health, West China Second University Hospital, Sichuan University, Chengdu, China; ^6^Sichuan Birth Defects Clinical Research Center, West China Second University Hospital, Sichuan University, Chengdu, China

**Keywords:** neutropenic diet, infection, mortality, meta-analysis, oncology

## Abstract

**Background:**

The purpose of this systematic review and meta-analysis was to evaluate the effect of a neutropenic diet and a control diet on infection and mortality rates in oncology patients with neutropenia.

**Methods:**

We searched the following English electronic databases: PubMed, Embase, Cochrane Central Register of Controlled Trials, and Google Scholar Engine. Published studies involving neutropenic diets (study group) and control diets (control group) in oncology patients with neutropenia were searched. The focus of the meta-analysis was on the outcomes of infection and mortality rates. A subgroup analysis was also performed.

**Results:**

A total of 6 studies were included, with a total sample size of 1114 patients. The patients in the study group had a similar infection rate compared with the patients in the control group (P = 0.11). The patients in the study group had a similar mortality rate compared with the patients in the control group (P = 0.74). Another subgroup analysis showed that the incidence of infection was also similar for pediatric (P = 0.74) and adult (P = 0.11) oncology patients between the study and control groups.

**Conclusions:**

Based on the current evidence, this meta-analysis showed that the application of a neutropenic diet cannot reduce the risk of infection and mortality in oncology patients with neutropenia. However, more rigorous randomized controlled trials are needed to confirm this conclusion in the future.

## Introduction

In recent years, the long-term survival rate of oncology patients has gradually increased through multimodal treatment, including chemotherapy (1–3) and radiation therapy (4, 5). However, in these patients receiving intensive chemotherapy and radiation therapy, immune function (6, 7) and bone marrow cells are inhibited (8, 9), resulting in long-term neutropenia (10–12). Destruction of the gastrointestinal mucosa also increases the risk of gastrointestinal infections in tumor patients (13, 14). At the same time, it is understood that patients with cancer may experience dysbiosis or an imbalance between potentially beneficial and pathogenic bacteria (15–17) due to exposure to broad-spectrum antibiotics and certain antitumor agents, as well as changes in intestinal peristalsis and integrity (18, 19). Therefore, it also potentially increases the risk of infection in these patients.

A variety of management and treatment protocols have been reported to effectively reduce infections in neutropenia. As previously reported, a “low bacterial diet” or “neutropenic diet” with varying degrees of dietary restriction was implemented to reduce the bacterial load available for infection (20–22). Although there is no evidence of consistency, the “neutropenic diet” is still widely used to treat cancer patients. However, given that malnutrition is a common side effect of cancer patients (23, 24), dietary restrictions may not be well tolerated, and it is also one of the important factors affecting the negative quality of life of patients.

The “neutropenic diet” was initially used as a therapeutic strategy to reduce the risk of acquiring systemic infection in patients with severe immunodeficiency, theoretically limiting patient intake of potential pathogenic microbes (25, 26). Although extensively recommended by oncologists, there are very little clinically relevant data to support this intervention to reduce the effects of chemotherapy-induced leukopenia in cancer patients. To date, a “neutropenic diet” has still no standard definition. Furthermore, to our knowledge, there have been few meta-analyses comparing the “neutrophil diet” and standardized dietary guidelines in leukemia patients with neutropenia, and the benefits and clinical outcomes in oncology patients with neutropenia treatment are unclear.

Thus, we performed a systematic review and meta-analysis and compared the efficacy of neutropenic diets in oncology patients with neutropenia. We hypothesized that (1) the neutropenic diet reduces the incidence of infection and (2) the neutropenic diet reduces the incidence of mortality.

## Materials and Methods

### Search Strategy

PubMed (1966 to October 2021), Embase (1974 to October 2021), Web of Science (1990 to October 2021), and Central Register of Controlled Trials (October 2021). To search for additional eligible studies, we also used Google Scholar Engine (October 2021). All comparative studies were involved in oncology patients with neutropenia. The following keywords were used: “bone marrow transplant”, “leukemia”, “neutropenic diet”, and “low bacterial diet”. No language and geographic restrictions.

### Inclusion Criteria

The meta-analysis used the following inclusion criteria: PICOS (population, intervention, comparator, outcome, study design). (1) Population: the patients were diagnosed with leukemia with neutropenia, including acute lymphoblastic leukemia (AML) and acute myeloid leukemia (ALL); (2) intervention: the intervention was a neutropenic diet (study group); (3) comparison: the comparator was a standardized diet (control group); and (4) results: the incidence of infection and mortality rates. (5) Study design: The study design was a clinical controlled trial. Duplications of published literature, letters, and abstracts were excluded. If the published study was repeated, the study with the largest sample size was retained. All titles and abstracts were independently reviewed by two reviewers. Any discrepancies were resolved by consensus, and if necessary, a third reviewer was consulted.

### Assessment of Methodological Quality

The methodological qualities of the included trials were assessed independently by the two reviewers. On the one hand, randomized controlled trials used a specific tool to assess the methodological quality and risk of bias of clinical trials described by the Cochrane Collaboration for Systematic Reviews (27). The seven items of random sequence generation, allocation sequence concealment, blinding of participants and personnel, blinding of the outcome assessment, incomplete outcome data, selective reporting, and other biases contained in the specific tool were meaningful evaluation indices. The overall methodological quality of each included study was evaluated as “Yes” (low risk of bias), “No” (high risk of bias), or “Unclear” (unclear risk of bias). On the other hand, for case–control study, the Newcastle–Ottawa Scale quality assessment scale was selected, which was recommended by the Cochrane nonrandomized studies methods (28).

### Data Extraction and Outcome Measures

Two reviewers extracted the results independently from the included studies, including the following information: first author, date of publication, number of participants, type of disease, method of intervention, antibiotic management, and outcome measures. The outcome measures used in this meta-analysis were the infection rate and survival rate between the study group and control group.

### Data Synthesis

The statistical analysis of all eligible studies was performed by RevMan 5 software (Version: 5.3, The Cochrane Collaboration), and a p value of < 0.05 was considered statistically significant. For continuous data, the mean difference and 95% confidence interval (CI) were calculated. For dichotomous data, the risk ratio (RR) and 95% CI were calculated. The meta-analysis assessed statistical heterogeneity using the chi-squared test and I^2^ statistic. According to the meaning of statistical heterogeneity, the fixed-effects model was selected if the chi-squared test > 0.1 or the I^2^ < 50%. Otherwise, the random-effects model was used to analyze the pooled data. Publication bias was assessed using a funnel plot of infection rates.

## Results

### Search Program

**Figure 1:**
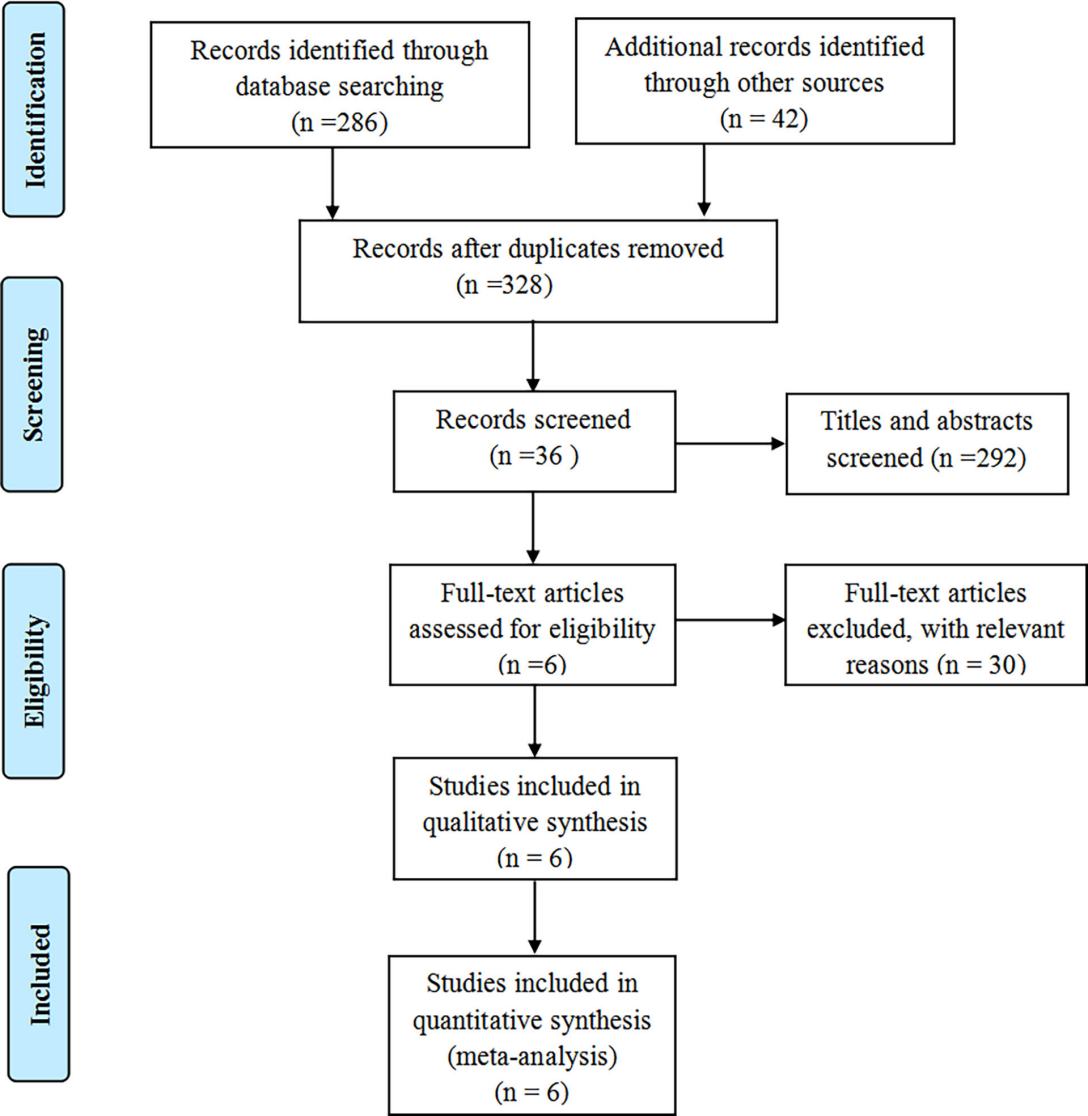
The flow diagram of literature selection.

**Figure 1** shows the flow chart for the inclusion and exclusion of the study. According to the inclusion criteria, 6 studies (20–22, 29–31) were finally included.

### Study Characteristics and Quality Assessment

The meta-analysis included 562 patients in the study group and 552 patients in the control group. Sample sizes ranged from 9 to 363. All included studies were published between 2007 and 2017. The average age was in the range of 4.1 to 77 years old. Two studies (20, 31) were reported for pediatric oncology patients, and 4 studies (21, 22, 29, 30) were reported for adult oncology patients with neutropenia. The “neutropenic diet” in the included studies has different definitions in various studies, including the elimination of raw fruits and vegetables. The “standardized diet” in the control group was either a “regular hospital diet” or a diet based on the “Food and Drug Administration (FDA)” approved food safety guidelines. The disease types in most studies were AML (21, 22, 29–31) and ALL (20, 21, 29–31). Four studies (21, 22, 29, 30) reported that patients received antibiotics to prevent infection. **Table 1** shows the baseline characteristics of all included studies and the **Table 2** shows the intervention and the outcome of all included studies.

**Table 1:** The baseline characteristics of all included studies.

First author	Date (y)	Country	Age (y)		Number of participants (n)		Type of disease
			Study group	Control group	Study group	Control group	
Moody et al. (20)	2006	United States	4.4	4.1	9	10	ALL, Osteosarcoma, Ewing’s sarcoma, Medulloblastoma
van Tiel et al. (21)	2007	Netherlands	51.8	53.3	10	10	ALL, AML
Gardner et al. (22)	2008	United States	64	63	78	75	AML, MDS
Trifilio et al. (29)	2012	United States	57	57	363	363	ALL, AML, Myeloma, Non-Hodgkin lymphoma, Hodgkin’s disease
Lassiter et al. (30)	2015	United States	45	45	25	21	ALL, AML, Non-Hodgkin lymphoma
Moody et al. (31)	2018	United States	12	11	77	73	ALL, AML, CNS tumors, non-CNS solid tumors, and carcinoma

y, years; n, number; ALL, Acute lymphocytic leukemia; AML, acute myeloid leukemia; MDS, myelodysplastic syndrome; CNS, central nervous System.

**Table 2:** The intervention and the outcome of all included studies.

First author	Intervention		Antibiotic prevention	Outcome measures	NOS scores
	Study group	Control group			
Moody et al. (20)	Patients received the neutropenic diet	Patients received FDA-approved food safety guidelines	Not Stated	Infection	–
van Tiel et al. (21)	Patients received low bacterial diet	Patients received normal hospital diet	Ciprofloxacin(500 mg every 12 h, orally) and fluconazole (50 mg every 24 h, orally)	Infection	–
Gardner et al. (22)	Patients received no raw fruits or vegetables (cooked diet)	Patients received a diet containing fresh fruit and fresh vegetables (raw diet)	Levofloxacin,valacyclovir, itraconazole, voriconazole, amphotericin B	Infection and Mortality	–
Trifilio et al. (29)	Patients received a neutropenic diet	Patients received a general hospital diet	Ciprofloxacin, a triazole antifungal, and acyclovir	Infection and Mortality	6
Lassiter et al. (30)	Patients received a neutropenic diet	Patients received a diet without restrictions	Ciprofloxacin 750 mg by mouth twice per day and metronidazole 500 mg by mouth three times per day, acyclovir 400 mg by mouth twice per day.	Infection	–
Moody et al. (31)	Patients received the neutropenic diet	Patients received FDA-approved food safety guidelines	Not Stated	Infection	–

FDA, Food and Drug Administration; NOS, Newcastle-Ottawa Scale.

**Figure 2:**
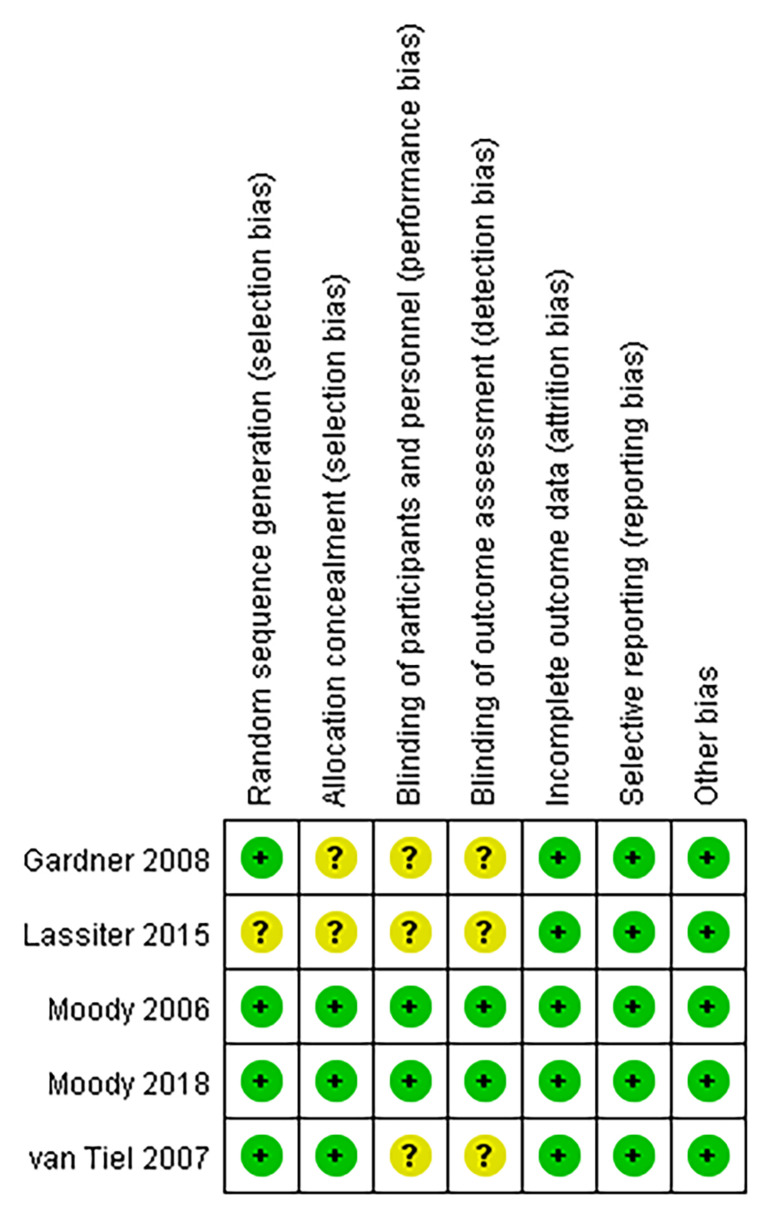
Risk of publication bias summary.

**Figure 3:**
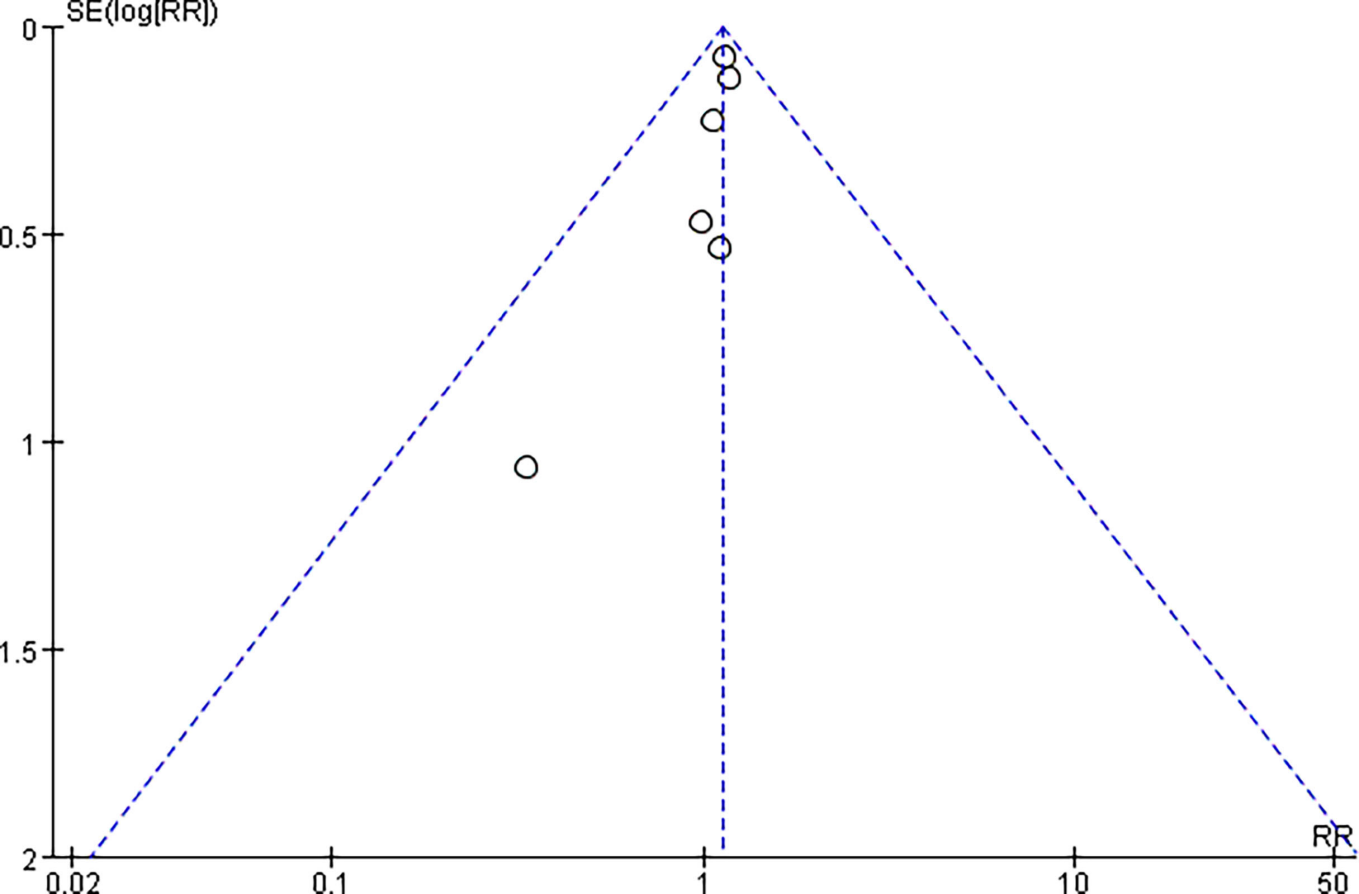
Publication bias of the infection.

**Figure 2** summarizes the methodological quality, and all studies were relatively well designed. Randomized methods were performed in 4 studies (20–22, 31), and 2 randomized controlled trials (20, 31) reported clear blinding. The Newcastle–Ottawa Scale was awarded higher scores, with 6 for the case–control study (29) (**Table 2**). The meta-analysis independently used funnel plots of the infection rates to assess any publication bias; the plots were generally symmetrical and suggested a low publication bias (**Figure 3**).

### Meta-Analysis of the Incidence of Infection

**Figure 4:**
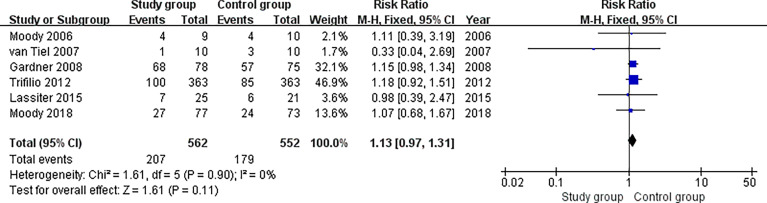
Meta-analysis of the incidence of infection.

All the included studies (20–22, 29–31) reported relevant data on the incidence of infection (562 and 552 patients in the study and control groups, respectively). The meta-analysis found that patients in the study group had similar results for the incidence of infection compared with the patients in the control group (risk ratio, 1.13; 95% CI, 0.97 to 1.31; P = 0.11). The fixed effects model was adopted in the meta-analysis since the pooled data did not show statistical heterogeneity (P = 0.90, I^2^ = 0%) (**Figure 4**).

### Subgroup Analysis for Pediatric and Adult Oncology Patients With Neutropenia

**Figure 5:**
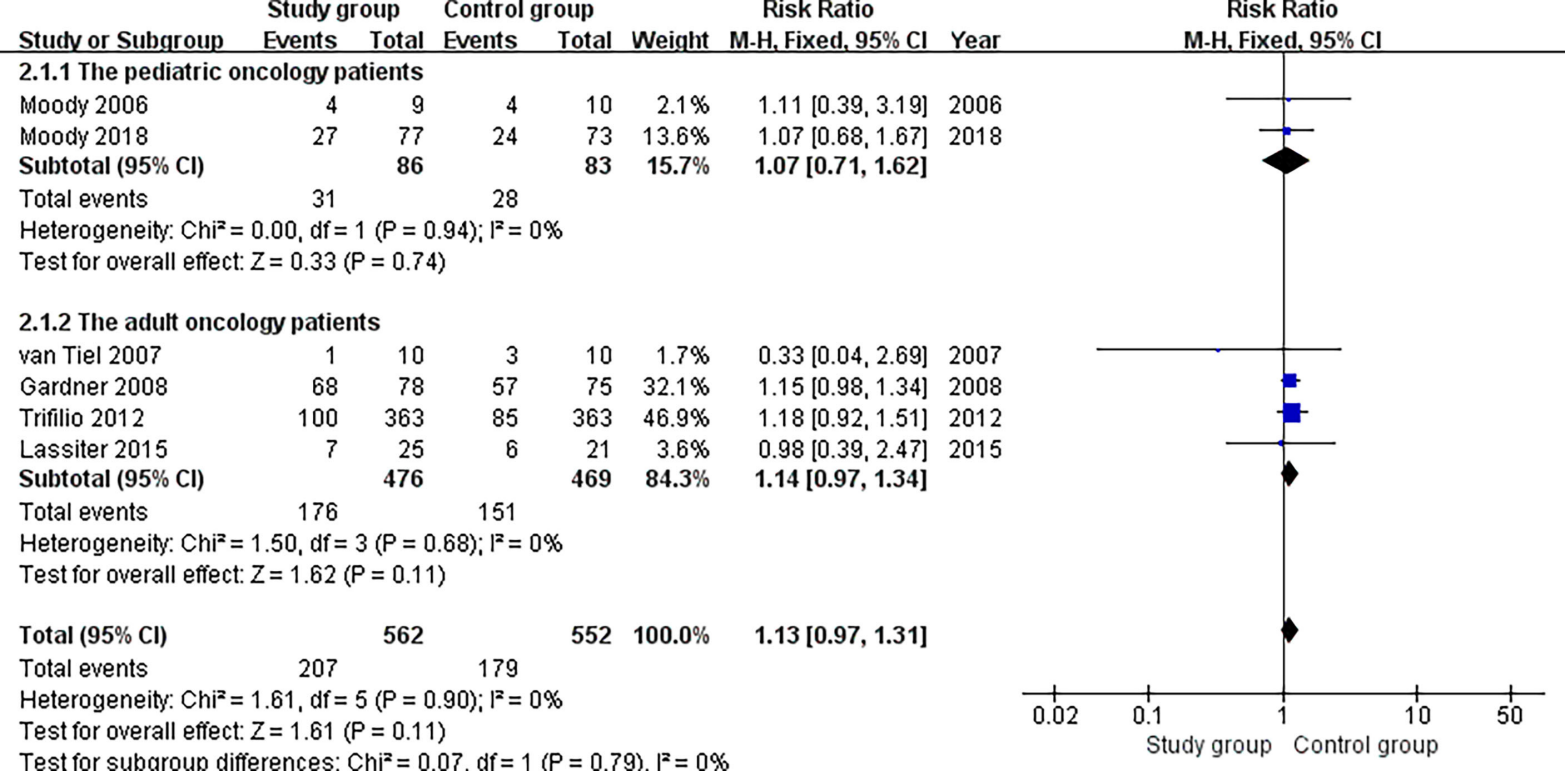
Subgroup analysis for pediatric and adult oncology patients.

Two studies (20, 31) reported relevant data on the incidence of infection for pediatric oncology patients (86 and 83 patients in the study and control groups, respectively). The subgroup analysis found that patients in the study group had similar results for the incidence of infection compared with the patients in the control group (risk ratio, 1.07; 95% CI, 0.71 to 1.62; P = 0.74). The fixed effects model was adopted in the meta-analysis since the pooled data did not show statistical heterogeneity (P = 0.94, I^2^ = 0%) (**Figure 5**).

Four studies (21, 22, 29, 30) reported relevant data on the incidence of infection for adult oncology patients (476 and 469 patients in the study and control groups, respectively). The subgroup analysis found that patients in the study group had similar results for the incidence of infection compared with the patients in the control group (risk ratio, 1.14; 95% CI, 0.97 to 1.34; P = 0.11). The fixed effects model was adopted in the meta-analysis since the pooled data did not show statistical heterogeneity (P = 0.68, I^2^ = 0%) (**Figure 5**).

### Meta-Analysis of the Incidence of Mortality

**Figure 6:**
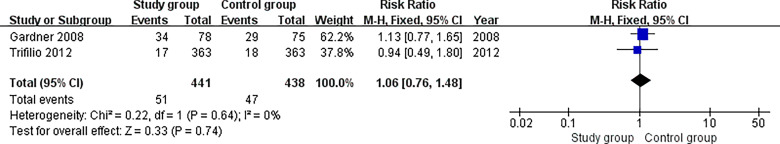
Meta-analysis of the incidence of mortality.

Two studies (22, 29) reported relevant data on the incidence of mortality (441 and 438 patients in the study and control groups, respectively). The meta-analysis found that patients in the study group had similar results for the incidence of mortality compared with the patients in the control group (risk ratio, 1.06; 95% CI, 0.76 to 1.48; P = 0.74). The random effects model was adopted in the meta-analysis since the pooled data did not show statistical heterogeneity (P = 0.64, I^2^ = 29%) (**Figure 6**).

## Discussion

This is a systematic review and meta-analysis to demonstrate whether neutropenic diets in oncology patients with neutropenia can reduce infection compared to a standardized diet. The meta-analysis found that a “neutropenic diet” did not reduce the incidence of infection and mortality in patients compared with a standardized diet. In addition, a subgroup analysis found no difference in the incidence of infection for neutropenia diets between children and adults.

Oncology patients with neutropenia who are affected by myelosuppressive chemotherapy cause neutropenia and are often instructed to follow “neutropenic diets” to reduce the risk of target infection and death (22, 29). However, “neutropenic diets” often interfere with patient food choices and may result in inadequate nutrient intake during chemotherapy. Previous studies (20, 21, 29, 31) have been extensively published on the application of a “neutropenic diet” in leukemia patients, and it has produced inconsistent clinical results. A randomized controlled trial (31) involving 150 patients who had AML, ALL or a malignant solid tumor and will undergo a period of myelosuppressive chemotherapy indicated that patients receiving a “neutropenic diet” showed no difference in the prevention of infection in pediatric oncology patients compared with those who received FDA-approved food safety guidelines (33% vs. 35%, P = 0.78). Similarly, another randomized controlled trial (20) of 19 patients comparing patients who received a neutropenic diet versus an FDA diet in pediatric oncology patients receiving myelosuppressive chemotherapy indicated that both a neutropenic diet and an FDA diet were similar in preventing infection (44% vs. 40%). In contrast, Trifilio et al. (29) showed an increased risk of infection in patients with a “neutropenic diet” and a higher risk of developing diarrhea and urinary tract infections. This may be a change in the microbiota and normal gut flora following the use of a “neutropenic diet”, as these infections have been shown to be associated with changes in bacterial volume.

The meta-analysis also raises questions about whether neutropenic patients have restricted diets, and this is especially important for cancer patients who have important problems with cachexia and anorexia (32). Fresh vegetables and fruits are an important source of dietary fiber, and fiber is important for improving intestinal transport and reducing constipation. Therefore, if these fibrous diets are excluded, they are harmful to the body to some extent. The results of this meta-analysis showed that the infection rate of patients with a neutropenic diet was similar to that of the FDA-approved food safety guidelines, which may suggest that oncologists should reconsider whether a neutropenic diet should be treated as a routine treatment.

The subgroup analysis in the meta-analysis also showed that in oncology patients with neutropenia in pediatric patients and adults, a “neutropenic diet” does not reduce the risk of infection. This is important for children and adults with cancer, as food restrictions can have a negative impact on their quality of life. Therefore, a standardized diet rather than a “neutropenic diet” is preferred because it is less restrictive, making it easier for them to meet calorie and survival needs. In addition, previously published studies (29, 31) have shown that standardized diets are more likely to freely access food than neutropenic diets and are also beneficial in saving the cost of medical care for inpatients.

However, it is still unclear whether reducing food restrictions increases the quality of life of cancer patients. Moody et al. (20) used the Peds QL Quality of Life Inventory Core Module and Cancer Module to measure health-related quality of life and found that the average score for the baseline core module was 73 for patients in the food safety group and 55 for patients in the neutropenia diet group. This difference was statistically significant in both groups, indicating poor quality of life in patients fed a neutropenia diet. However, Hamada et al. (33) showed that the management of dietary restrictions did not directly reduce the quality of life in patients with hematological malignancies. Although the diets in the two groups are still controversial, studies on cancer patients in adults and children have shown that dietary intake is an important factor in quality of life and is closely related to physical health. Therefore, proper diet management and selection require further research to identify the best program to improve the quality of life of patients.

Another interesting question is whether granulocyte colony stimulating factor (GCSF) can affect the incidence of infection. We reviewed 6 studies and found that a total of 3 studies reported the use of GCSF. Gardner et al. (22) reported CGSF, but there was no exact number of reports used. Moody et al. (20) reported CGSF in the study group (4/9) and the control group (5/10), but the infection results were not significantly different. Moody et al. (31) reported CGSF in the study group (43/77) and the control group (40/73), and the infection was not significantly different. Although we wanted to use a meta-analysis to assess the impact of GCSF use on infection outcomes in this study, it could not be included in the meta-analysis calculation considering that not all patients were using GCSF. However, in terms of infection rates in each study, the use of GCSF seems to have little impact on infection rates, but it is still necessary to verify the results of many samples in the future due to the limited sample size.

The meta-analysis also has some limitations. First, the main limitation is its methodological quality. Randomized controlled trials are the most effective and reliable study to evaluate clinical outcomes in meta-analysis, but we included a case–control study that may produce quality bias and affect the results. Therefore, more carefully and scientifically designed randomized controlled trials are needed in the future to further confirm and compare the differences between neutropenic diets and standardized diets. The second limitation of this study is the sample size. A larger sample is needed and will be able to answer whether it is safe to reduce the diet that limits neutropenia and follow the food safety guidelines for patients with neutropenia.

## Conclusion

Based on the current evidence, this meta-analysis showed that the application of a neutropenic diet does not reduce the risk of infection and mortality in oncology patients with neutropenia. However, more rigorous randomized controlled trials are needed to confirm this conclusion in the future.

## Data Availability Statement

The datasets presented in this study can be found in online repositories. The names of the repository/repositories and accession number(s) can be found in the article/supplementary material.

## Author Contributions

YM collects data and writes manuscript. XL conducted statistical analysis and designed experiments. HL designed experiments and revised manuscripts. All authors contributed to the article and approved the submitted version.

## Funding

The study was supported by the Science and Technology Department of Sichuan Province (2017FZ0056), and it was supported by the West China Second University Hospital of Sichuan University (KX144).

## Conflict of Interest

The authors declare that the research was conducted in the absence of any commercial or financial relationships that could be construed as a potential conflict of interest.

## Publisher’s Note

All claims expressed in this article are solely those of the authors and do not necessarily represent those of their affiliated organizations, or those of the publisher, the editors and the reviewers. Any product that may be evaluated in this article, or claim that may be made by its manufacturer, is not guaranteed or endorsed by the publisher.
